# Supplementary material for: Improving the 3D Printability and Mechanical Performance of Biorenewable Soybean Oil-Based Photocurable Resins
Source: Polymers (Basel). 2024 Apr 3;16(7):977. doi: 10.3390/polym16070977 (PMC11013316; doi:10.3390/polym16070977)
Supplement: Supplementary file 1 [file polymers-16-00977-s001.zip › polymers-2918897-supplementary.pdf]

polymers

MDPI

Supporting information

Improving the 3D printability and mechanical performance of biorenewable soybean oil-based photocurable resins

Marius BODOR[1], Aurora LASAGABÁSTER-LATORRE[2], Goretti ARIAS-FERREIRO[1,†], M. Sonia DOPICO-GARCÍA[1], María-José ABAD[1,*]

[1] Campus Industrial de Ferrol, Grupo de Polimeros-CITENI, Universidade da Coruña, Spain; marius.bodor@udc.es (M.B.); s.dopico@udc.es (S.D.-G.); goretti.arias@udc.es (G.A.-F.); mjabad@udc.es (M.H.-A.)

[2] Dpto Química Orgánica I, Facultad de Óptica y Optometría, Universidad Complutense de Madrid, Spain; aurora@quim.ucm.es

† Present address: Center for Cooperative Research in Biomaterials (CIC biomaGUNE), Basque Research and Technology Alliance (BRTA), Paseo de Miramón 194, 20014 Donostia San Sebastián, Spain

* Correspondence: mjabad@udc.es

S3. Results

S3.1. Viscosity of the liquid formulations

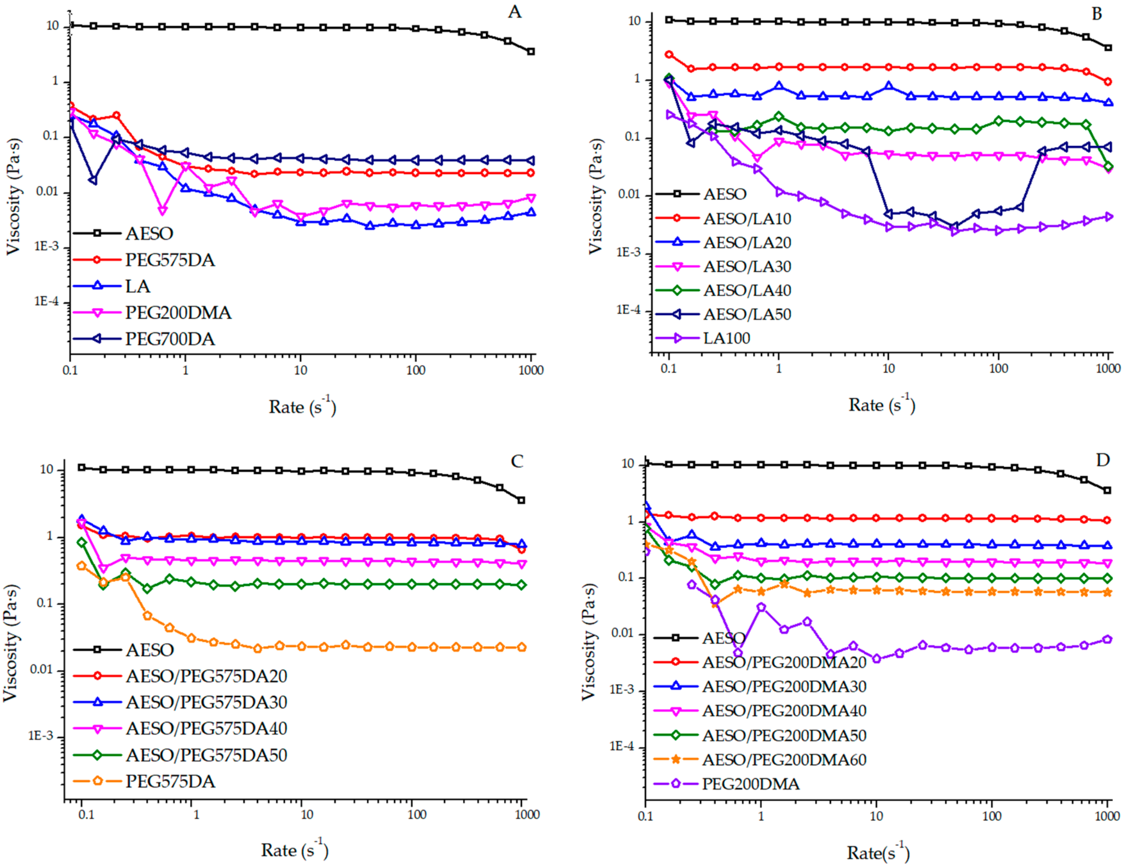

Figure S1. Viscosity values of (A) pure AESO and reactive diluents, (B) and mixtures of AESO/LA, (C) AESO/PEG575DA and (D) AESO/PEG200DMA as a function of shear rate at room temperature.

S3.2. Characterization of printed formulations

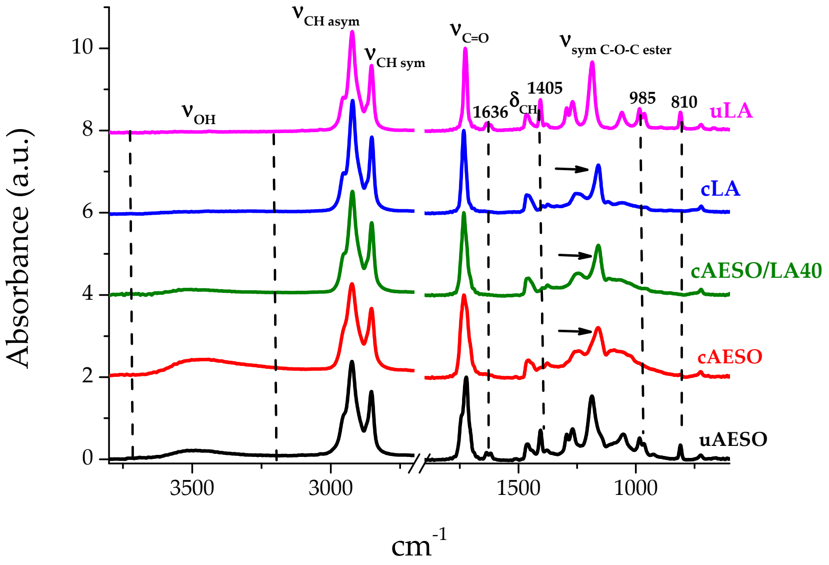

Figure S2. FTIR-spectra of uncured (u) and cured (c) AESO, LA and AESO/LA40. The dashed lines point at the bands assigned to the acrylate groups that disappear upon polymerisation. The arrows show the shift of the C-O-C stretching vibrations of the acrylate groups after polymerisation.

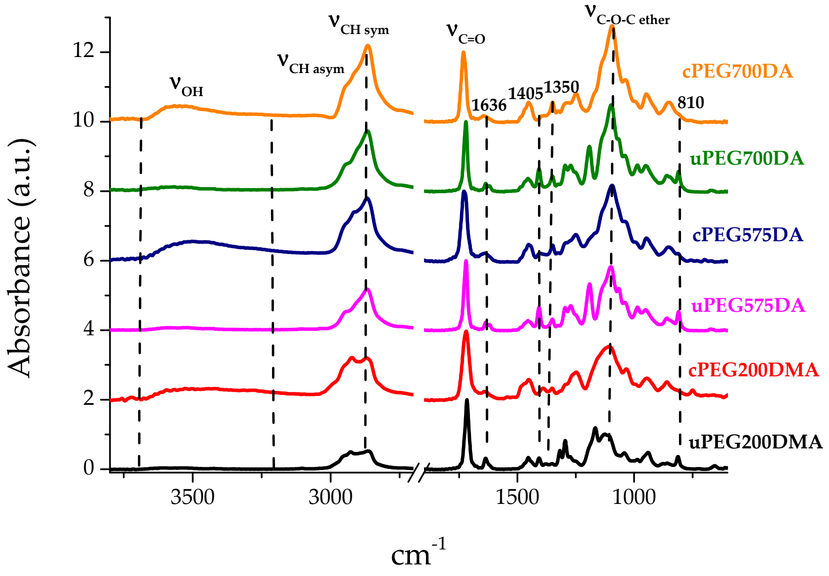

Figure S3. FTIR-spectra of uncured (u) and cured (c) PEG200DMA, PEG575DA and PEG700DA. The dashed lines point at the bands assigned to the acrylate groups that disappear upon polymerisation.

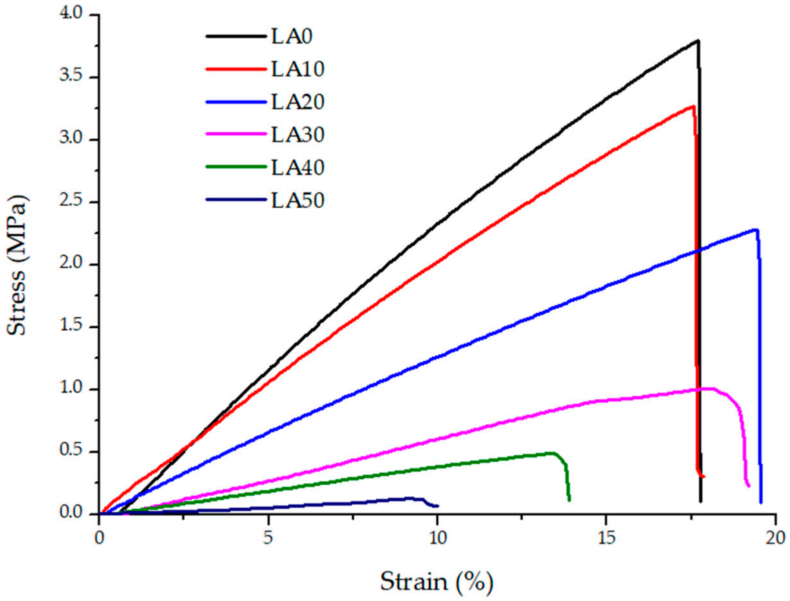

Figure S4. Representative strain-stress curves of AESO and AESO/LA copolymers.

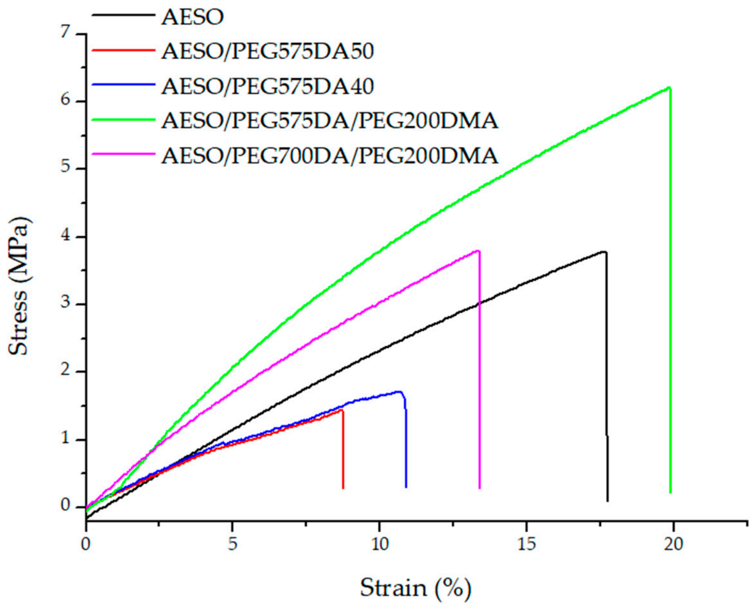

Figure S5. Representative strain-stress curves of AESO and AESO/PEGDA copolymers and terpolymers.

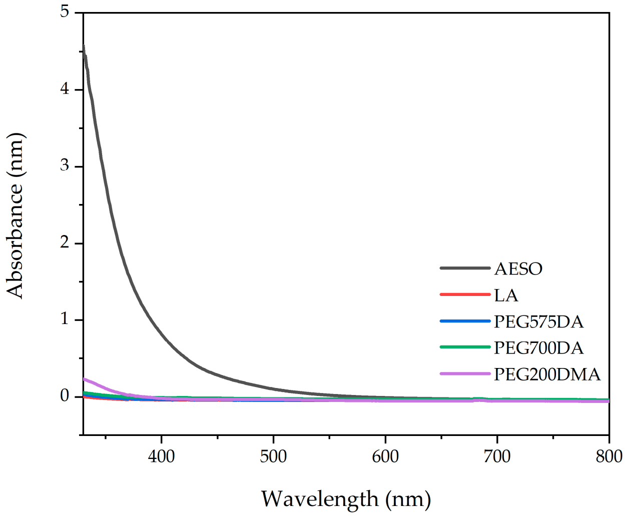

Figure S6. UV absorption spectra of the monomers used in formulations.
